# Unravelling the rate of action of hits in the *Leishmania donovani* box using standard drugs amphotericin B and miltefosine

**DOI:** 10.1371/journal.pntd.0005629

**Published:** 2017-05-25

**Authors:** Diana Tegazzini, Juan Cantizani, Imanol Peña, Julio Martín, Jose M. Coterón

**Affiliations:** Kinetoplastids DPU, Diseases of the Developing World (DDW), GlaxoSmithKline, Tres Cantos, Madrid, Spain; Northeastern University, UNITED STATES

## Abstract

In recent years, the neglected diseases drug discovery community has elected phenotypic screening as the key approach for the identification of novel hit compounds. However, when this approach is applied, important questions related to the mode of action for these compounds remain unanswered. One of such questions is related to the rate of action, a useful piece of information when facing the challenge of prioritising the most promising hit compounds. In the present work, compounds of the “*Leishmania donovani* box” were evaluated using a rate of action assay adapted from a replicative intracellular high content assay recently developed. The potency of each compound was determined every 24 hours up to 96 hours, and standard drugs amphotericin B and miltefosine were used as references to group these compounds according to their rate of action. Independently of this biological assessment, compounds were also clustered according to their minimal chemical scaffold. Comparison of the results showed a complete correlation between the chemical scaffold and the biological group for the vast majority of compounds, demonstrating how the assay was able to bring information on the rate of action for each chemical series, a property directly linked to the mode of action. Overall, the assay here described permitted us to evaluate the rate of action of the “*Leishmania donovani* box” using two of the currently available drugs as references and, also, to propose a number of fast-acting chemical scaffolds present in the box as starting points for future drug discovery projects to the wider scientific community. The results here presented validate the use of this assay for the determination of the rate of action early in the discovery process, to assist in the prioritisation of hit compounds.

## Author summary

Visceral leishmaniasis is a parasitic disease, caused by *Leishmania donovani* and *Leishmania infantum* species, found in Asia, South America, East Africa and Southern Europe. Currently available treatments present either serious side effects or high prices and, therefore, the identification of a new generation of antileishmanial drugs is clearly an unmet medical need. In order to find new and safer drugs, pharmaceutical companies and research institutes have engaged in screening of large compound collections on cell-based systems. These assays are more likely to predict the *in vivo* potency of the hits identified compared to assays based on the inhibition of a specific target, but do not provide any information regarding one of the most important parameters, together with PK/PD profile, used to predict the effect of a drug as it is the mode of action. In addition, most of the available assays rely on a single endpoint determination of compound potency and, therefore, do not evaluate rate of action, a property linked to the mode of action. Although there is only limited knowledge on the PK/PD profile for current antileishmanial drugs amphotericin B and miltefosine, the observation that *in vitro* fast-acting amphotericin B requires shorter treatment regimen than *in vitro* slow-acting drug miltefosine seems to suggest that this property could be very useful for progressing the most promising hit compounds to more advanced stages of the drug discovery process. In fact, progression of fast-acting compounds over slow-acting ones is the current strategy at the Kinetoplastid DPU. For these reasons, we present here the application of a rate of action assay to a promising compound set displaying potencies against the intracellular *Leishmania donovani* parasite (”*Leishmania donovani* box”) in the micromolar to sub-micromolar range as well as its validation for use in the hit prioritisation within drug discovery.

## Introduction

*Leishmania* parasites are causative agents of different pathologies, ranging from self-curing but disfiguring cutaneous leishmaniasis (CL) to visceral leishmaniasis (VL), mainly caused by *L*. *donovani* species in humans and potentially lethal if untreated. The few treatments available for VL, including amphotericin B (AB) and miltefosine (MF), are all characterized by important drawbacks that underline the urgent need for new drugs [1]. For example, amphotericin B showed successful cure rates after a single-dose administration [2]. However, it requires intravenous administration, patient hospitalization and unbroken cold chain that, together with the high cost of the liposomal formulation, make it far from ideal for usage in endemic countries [3]. Miltefosine, the only oral drug available to treat visceral leishmaniasis, is associated to teratogenicity and requires a long treatment course of at least 28 days, which represents a huge obstacle to patient’s compliance [4].

For the last 25 years, drug discovery focused primarily on target-based approaches [5], that rely on the ability of a drug to exert its effect through inhibition or interference with one or more essential targets or pathways. These approaches always require an important initial effort for the identification of the right targets, as their success is dependent on target essentiality and druggability [6]. They are even more challenging for the kinetoplastids area due to the small number of drug targets fully validated in the field [7,8], and also to the lack of knowledge about mode of action for currently available drugs, which potency seems to reflect the inhibition of multiple targets or pathways [9].

For all these reasons, over the last few years, drug discovery against kinetoplastid parasites has started to consider phenotypic screening [10,11], where compounds are tested directly on a cell-based system and compound potency is evaluated on the basis of the insurgence of a phenotype, which is usually death or reduced growth of the organism. One of the major limitations of phenotypic screenings is the lack of information on the molecular target or mode of action for the hits identified [12]. This gap also includes information on the rate of action, defined as the time needed to reach maximum effect under the same *in vitro* experimental conditions. This property can be inferred in many cases from the mode of action of promising hits, if known, or has to be specifically addressed otherwise. In fact, besides the filters traditionally applied to compound prioritisation in the drug discovery process (mainly physico-chemical properties and toxicity), knowledge of their rate of action would provide an exceptional piece of additional information. Recently, R&D efforts against other protozoan parasites like *P*. *falciparum*, *T*. *brucei* and *T*. *cruzi* have addressed the question of determining the rate of action of hit compounds to aid the selection of the most promising ones [13–17].

As part of the academia-industry partnership approach to defeat neglected tropical diseases, GSK has very recently conducted a high throughput screening campaign with ca. 1.8 million compounds belonging to the GSK compound collection against the three kinetoplastid parasites *Leishmania donovani*, *Trypanosoma cruzi* and *Trypanosoma brucei*, assembling three boxes of an average of 200 hit compounds each, active against these parasites [18]. Also, we have recently developed a high content *L*. *donovani* replicative intra-macrophage *in vitro* system that appears to be a promising translational infection model [19]. In the present work, we have further developed this assay to investigate its potential for the evaluation of compound rate of action, and analysed in depth the use of this property in the prioritization of hit compounds for early drug discovery programmes. To fully exploit the potential of the “*L*. *donovani* box” recently published [18], in the present work, we determined the potency of these compounds in the replicative intra-macrophage assay recently described [19], assessing the potency of each compound every 24 hours up to 96 hours. This screening allowed us to obtain time-to-kill information for each compound, to compare this information with amphotericin B and miltefosine, and to group them into three different categories according to rate of action. We also clustered the compounds according to their chemical structure and matched the results from the two exercises, demonstrating how this assay can be applied to the hit progression cascade in drug discovery.

## Materials and methods

### Cell lines

The human biological samples were sourced ethically following GSK-HBSM guideline and policies, their research use was in accord with the terms of the informed consents. THP-1 cells (human monocytic leukemia) were made available by GSK-Biological Reagents and Assay Development Department (GSK-BRAD, Stevenage, UK) and were maintained in RPMI media (Life-Technologies) supplemented with 1.25 mM Pyruvate (Life-Technologies), 2.5 mM Glutamine (Life-Technologies), 25 mM HEPES (Life-Technologies) and 10% heat inactivated FBS (Gibco).

The *Leishmania donovani* strain (MHOM/SD/62/1S-CL2D, LdBOB) [20] expressing green fluorescent protein (GFP) was kindly provided by Manu de Rycker (University of Dundee) [21]. Axenic amastigotes were grown at 37°C, 5% CO_2_ in media containing 15 mM KCl (Invitrogen^™^), 10 mM K_2_HPO_4_ (Merck), 136 mM KH_2_PO_4_ (Merck), 0.5 mM MgSO_4_ (Sigma-Aldrich), 24 mM NaHCO_3_ (Invitrogen^™^), 25 mM Glucose (Sigma-Aldrich), 1mM L-Glutamine (Invitrogen^™^), 1xRPMI Vitamin Solution (Sigma-Aldrich), 10 μM Folic Acid (Sigma-Aldrich), 100 μM Adenosine (Sigma-Aldrich), 5 mg/L Hemin (Sigma-Aldrich), 1xRPMI Amino Acid solution (Sigma-Aldrich), 25 mM MES, 0.0004% Phenol Red and 20% Heat Inactivated FBS (Gibco) in Milli-Q water (pH = 5,5 at 37°C). The selection antibody Lexsy NTC (Nourseothricin, Jena Bioscience) was regularly added to amastigote cultures [21].

### *In vitro* intra-macrophage *L*. *donovani* assay

The *L*. *donovani* intra-macrophage assay was adapted from previously described protocols [18, 19, 21], with the exception that four copies of compound plates were prepared to allow fixing and staining at different time points (24, 48, 72 and 96 hours). THP-1 cells were differentiated in 225 cm^3^ flasks (6x10^5^ cells/mL, 80 ml) with 30 nM of phorbol 12-myristate 13-acetate (PMA, Sigma-Aldrich). After 24 h incubation, the media containing PMA was removed, washing twice with complete growth media. Differentiated THP-1 cells were infected with a suspension of axenic amastigotes in complete THP-1 growth media without PMA (6x10^6^ parasites/mL, 80ml) for 24 hours. The media was subsequently removed and the monolayer washed with PBS. Cells were harvested with a solution of 0.25% (w/v) trypsin/EDTA in PBS, diluted to 1.6x10^5^ cells/mL in assay media containing RPMI media supplemented with 2% Heat Inactivated Horse Serum (Gibco), 25 mM NaHCO_3_ (Invitrogen^™^) and 30 nM PMA and dispensed in assay plates with a Multidrop Combi dispenser (Thermo Scientific). Uninfected differentiated THP-1 were treated following the same protocol and dispensed in each assay plate as highest compound response control. Assay plates were incubated at 37°C, 5% CO_2_ for 24, 48, 72 or 96 hours and then fixed with 4% formaldehyde for 30 min at room temperature (50 μl of 8% (v/v) formaldehyde solution (Sigma-Aldrich) in PBS/each well). Cells were then washed twice with 100 μL PBS using an EL406 multi well platewasher (BioTek), stained with 30 μl of a solution of DAPI (10μg/mL) and Triton X-100 (0.1% v/v) in PBS for 30 min at room temperature and washed twice with 50 μL PBS. Finally, 50 μL of PBS were added to each well, plates were sealed and stored at 4°C until analysis.

Four images per each well were acquired with a High-Content Screening System (Opera QEHS, Perkin Elmer) using a 20x air objective. Two sequential images were taken exciting at 405 nm (DAPI) and 488 nm (GFP). Macrophages were identified with DAPI staining while amastigotes were detected using the GFP signal. The images were analyzed with an image analysis algorithm developed on Acapella^®^ High Content Imaging and Analysis Software (PerkinElmer). THP-1 cell count (MAC), average number of amastigotes per macrophage (AM/MAC) and percentage of infected cells (INF) were calculated for each well.

### Test compounds and assay plates

Test compounds were made available by the GSK collection of compounds. Chemical structures and more information on all compounds tested in these studies are available at reference 18 as TCMDC IDs (Tres Cantos Medicine Discovery Center Identifiers). Amphotericin B (AB) and miltefosine (MF) were purchased from Sigma Aldrich.

Of the total 192 compounds of the “*L*. *donovani* box”, 176 compounds were assayed due to unavailability of 16 compounds at the time the work was carried out. Pre-dispensed 384-wells assay plates (Greiner μclear black) were prepared by adding 250 nL of compounds dissolved in 100% DMSO to each well by using an Echo^®^ liquid handler (Labcyte Inc.). Eleven-point one in three dilution curves were generated from a top concentration of 50 μM. Plates were stored at -20°C and allowed to equilibrate at room temperature before use.

### Data analysis

Data were normalized to percentage biological response following the equation:

$$\%Response=\frac{\left|R_{Ctrl1}-R_{x}\right|}{\left|R_{Ctrl1}-R_{Ctrl2}\right|}\cdot 100$$

where R_x_ is the assay response measured for each compound X, R_ctrl1_ the lowest response achieved in absence of any test compound (negative control) and R_ctrl2_ the highest response represented by non-infected cells (positive control). Control wells were included in each assay plate and R_ctrl1_ and R_ctrl2_ were calculated as the average of the replicates.

Activities were expressed as pEC50 (pEC50 = -log EC_50_ (M)). The pEC50 values at each time point for each compound were obtained using the ActivityBase XE nonlinear regression function in the full curve analysis bundle to fit the 4-parameter logistic equation.

### Analysis of pEC50 values

The pEC50 values of the compounds of the “*L*. *donovani* box” at each time point (24, 48, 72 and 96 hours) were calculated as described above using two outputs, AM/MAC and INF cells, and the mean of two independent experiments was used for the analysis. The two outputs were analyzed independently.

Only compounds showing pEC50 ≥5 at 96h, corresponding to *in vitro* activity values in the single-digit micromolar range or below (EC50 ≤10uM), were included in the analysis. Also, compounds showing poor fitting curves or low maximum asymptote (<60%) were excluded from the analysis. The list of remaining compounds was refined by applying the same filters applied for the assembly of the “*L*. *donovani* box” [18]: Selectivity Index (SI) at 96h versus THP-1 cells as primary toxicity determination (SI >5, pEC50_AM/MAC/INF_—pEC50_MAC_ >0.7) and versus HepG2 cells as secondary toxicity determination (SI >10, pEC50_AM/MAC/INF_—pEC50_HepG2_ >1). For those compounds fulfilling the requirements described, the difference in pEC50s between 96h and 24h (Δ_96h-24h_) and between 96h and 48h (Δ_96h-48h_) were calculated and compared to the same parameters obtained for AB and MF. According to their Δ_96h-24h_ and Δ_96h-48h_ values, each compound was assigned to one of the following “rate of action groups”: group 1 for compounds faster or similar to AB, group 2 for compounds faster or similar to MF but slower than AB, and group 3 for compounds slower than both AB and MF.

### Chemical clustering

The chemical structures of the high-quality compounds identified after application of potency and selectivity filters to initial hits were individually analysed and used to chemically cluster the compounds. A chemical scaffold was considered a cluster when it was present in at least three molecules of the compound set.

### Comparison between “rate of action” groups and chemical clusters

The compounds included in each chemical cluster described above were analyzed in terms of “rate of action” group from the biological clustering exercise. The expected outcome was that compounds belonging to a given chemical cluster should also be found in the same “rate of action” group.

## Results

### Assay characterization

**Fig 1:**
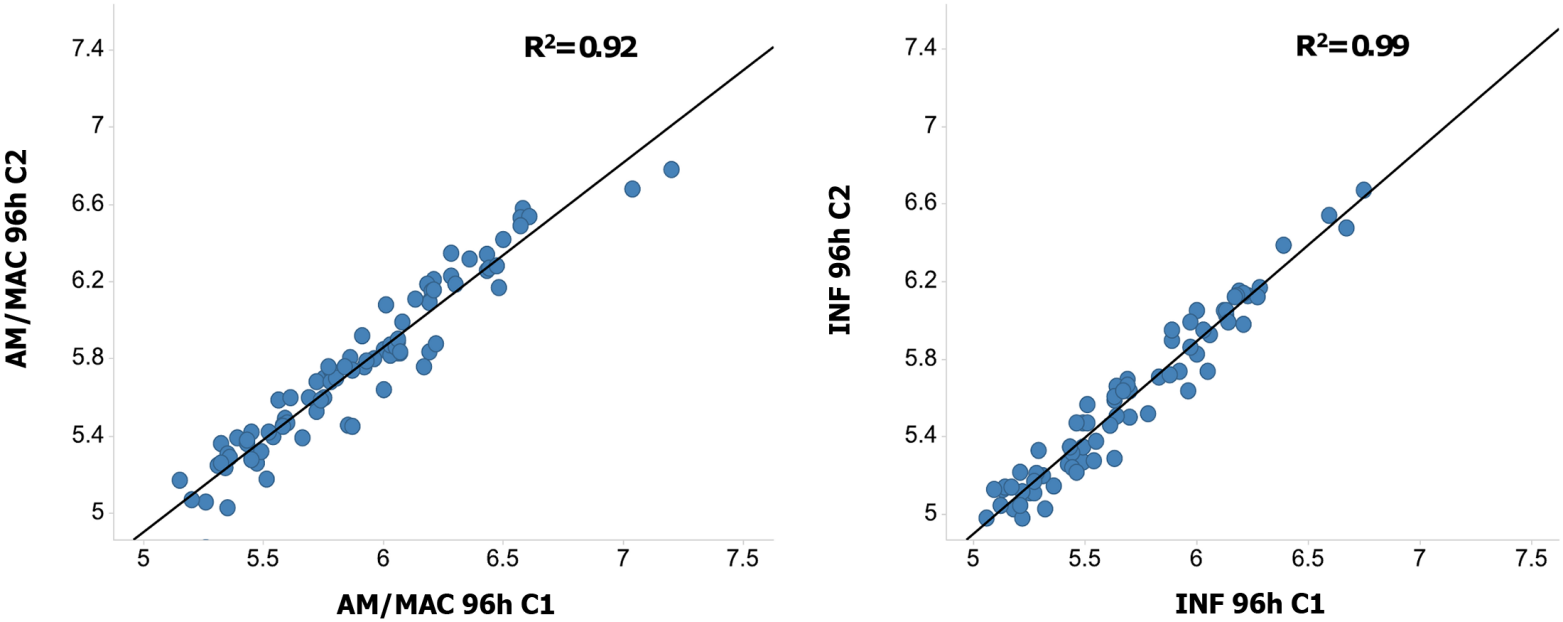
Comparison of the pEC50 values obtained at 96 hours for the two assay runs using the AM/MAC output (left) and the INF output (right). The R^2^ value calculated is shown at the top of each representation.

The mean pEC50 values (pEC50 = -log EC_50_ (M)) at each time point are shown in the supplementary material (S1 Table for AM/MAC output and S2 Table for INF output). The assay proved to be robust, with good to excellent Z prime values (S3 Table). The assay was also highly reproducible, showing R^2^ values> 0.9 when comparing the two copies for the two outputs used, AM/MAC and INF, as shown in Fig 1.

Table 1 shows pEC50 values as well as pEC50 differences obtained for standard drugs amphotericin B and miltefosine at each time point. pEC50 values obtained at 96h for both drugs were comparable to previously reported data in horse serum and foetal bovine serum [19, 21]. Amphotericin B reached its maximum effect in both outputs between 24 and 48 hours, while miltefosine reached its maximum effect clearly later than 48 hours.

**Table 1:** pEC50 values of standard drugs amphotericin B (AB) and miltefosine (MF) at each time point calculated using the AM/MAC and INF outputs. pEC50 = -log EC_50_ (M). Values are expressed as mean value of two independent test runs. R^2^ values obtained for all curves studied were >0.85.

**AM/MAC**	**pEC50**_**24h**_	**pEC50**_**48h**_	**pEC50**_**72h**_	**pEC50**_**96h**_	**Δ96h-24h**	**Δ96h-48h**
**AB**	6.87	7.11	7.27	7.16	0.29	0.05
**MF**	5.31	6.05	6.22	6.28	0.98	0.23
**INF**	**pEC50**_**24h**_	**pEC50**_**48h**_	**pEC50**_**72h**_	**pEC50**_**96h**_	**Δ96h-24h**	**Δ96h-48h**
**AB**	6.82	6.98	7.06	7.02	0.20	0.04
**MF**	5.16	5.68	6.19	6.20	1.04	0.52

### Compound selection for clustering and analysis

The selection of the most promising compounds amongst the initial 176 hits tested was done by application of a number of filters, including potency (pEC50>5), number of valid data points in the kinetic, THP-1 selectivity (SI>5), and HepG2 selectivity (SI>10). Two final compound sets, containing 88 and 73 high-value hits for the AM/MAC and INF outputs respectively, were obtained (S4 and S5 Tables) and subjected to chemical clustering and biological “rate of action” analysis.

### Analysis of AM/MAC output

The 88 selected compounds were divided into 3 groups according to their Δ_96h-24h_ and Δ_96h-48h_ values using as reference those values obtained for amphotericin B (0.29 and 0.05 respectively) and miltefosine (0.98 and 0.23 respectively). Group 1 included those compounds faster, similar or slightly slower than AB showing a Δ_96h-24h_<0.7. Group 2 was defined as those compounds showing Δ_96h-24h_>0.7 and Δ_96h-48h_<0.5, being faster, similar, or slightly slower than MF but clearly slower than AB. Finally, group 3, pooling compounds clearly slower than both AB and MF, was defined as hits showing Δ_96h-24h_>0.7 and Δ_96h-48h_>0.5. Applying these filters, the 88 compounds were grouped as shown in S6 Table: 64 compounds in group 1, 17 compounds in group 2 and 7 compounds in group 3.

**Fig 2:**
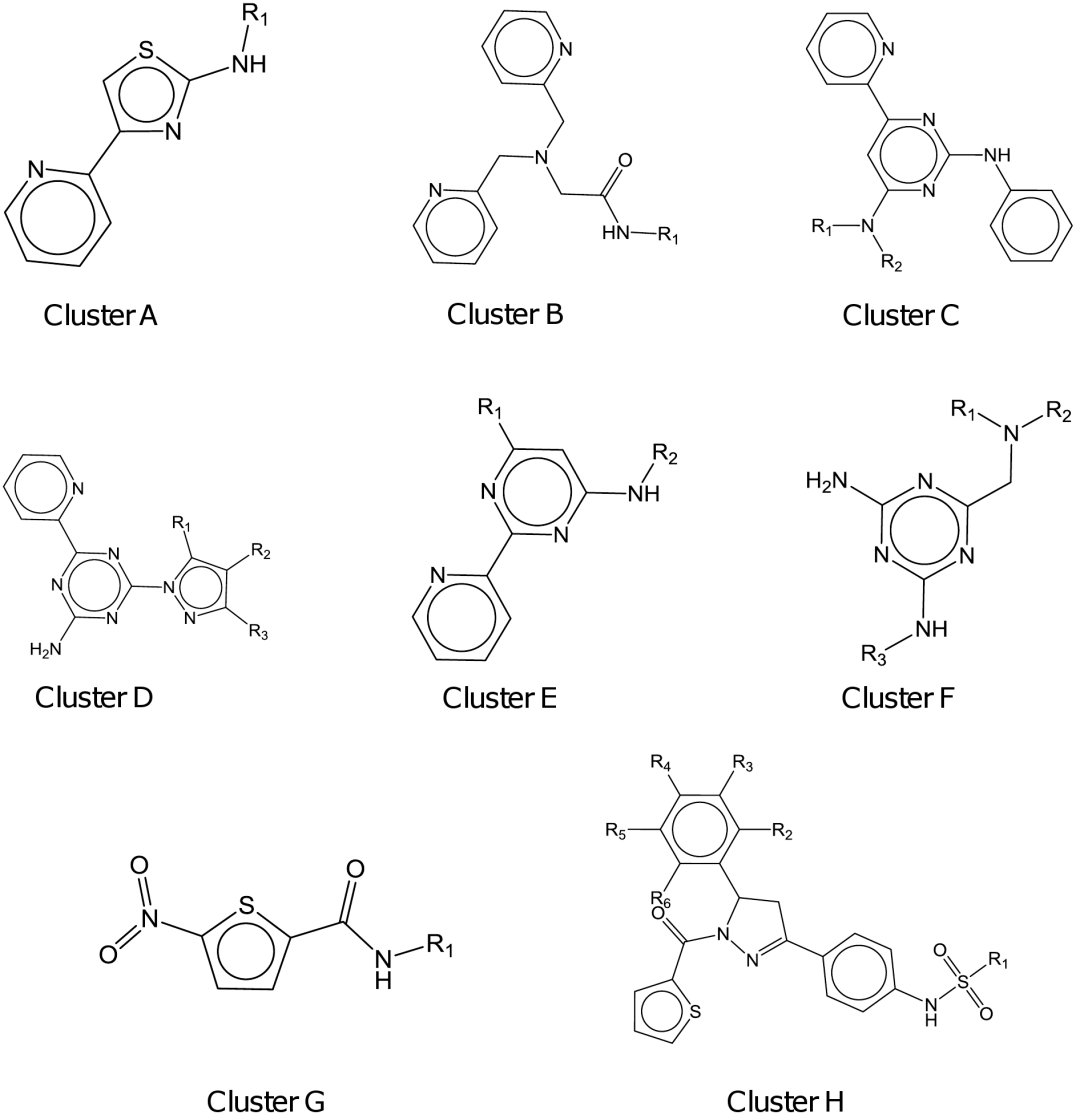
Complete set of chemical clusters obtained for the clustering exercise conducted with the 88 selected compounds using the data of the AMMAC output and the 73 selected compounds using the data of the INF output.

In parallel, the same 88 compounds were clustered according to the presence of a minimum common chemical scaffold. A set of seven clusters with at least 3 members totalling 35 compounds was obtained. The remaining 53 hits were either singletons or belonged to a cluster with less than 3 members. The chemical clusters obtained are shown in Table 2 and Fig 2.

**Table 2:** Chemical clusters (Cl) obtained for the 88 selected compounds with the AM/MAC output. TCMDC ID: Tres Cantos Medicine Discovery Center Identifier. Chemical structures and more information on all compounds tested in these studies are available at reference 18 as TCMDC IDs (Tres Cantos Medicine Discovery Center Identifiers).

	TCMDC ID	Cl		TCMDC ID	Cl		TCMDC ID	Cl	
	**143101**	**A**		**143093**	**B**		**143407**	**D**	
	**143113**	**A**		**143094**	**B**		**125826**	**E**	
	**143122**	**A**		**143095**	**B**		**143077**	**E**	
	**143133**	**A**		**143211**	**C**		**143078**	**E**	
	**143557**	**A**		**143212**	**C**		**143621**	**E**	
	**143558**	**A**		**143213**	**C**		**143144**	**F**	
	**143570**	**A**		**143214**	**C**		**143168**	**F**	
	**143584**	**A**		**143216**	**C**		**143427**	**F**	
	**143639**	**A**		**143217**	**D**		**143139**	**H**	
	**143090**	**B**		**143218**	**D**		**143140**	**H**	
	**143091**	**B**		**143404**	**D**		**143141**	**H**	
	**143092**	**B**		**143406**	**D**				

### Analysis of INF output

The 73 selected compounds were divided into 3 groups according to their Δ_96h-24h_ and Δ_96h-48h_ values using as reference those values obtained for amphotericin B (0.20 and 0.04 respectively) and miltefosine (1.04 and 0.52 respectively). In a similar way to the AM/MAC output, group 1 was defined as those compounds with Δ_96h-24h_<0.7. Group 2 was defined as those compounds showing Δ_96h-24h_>0.7 and Δ_96h-48h_<0.7. Group 3 was defined as hits showing Δ_96h-24h_>0.7 and Δ_96h-48h_>0.7. Applying these filters, we were able to group the compounds as follows: 49 compounds in group 1, 13 compounds in group 2 and 11 compounds in group 3 (S7 Table).

In parallel, the same 73 compounds were clustered according to the presence of a minimum common chemical scaffold. A set of seven clusters with at least 3 members comprising 34 compounds were obtained. The remaining 39 compounds were either singletons or belonged to a cluster with less than 3 members. Results can be found in Table 3 and Fig 2.

**Table 3:** Chemical clusters (Cl) obtained for the 73 selected compounds in the INF output. TCMDC ID: Tres Cantos Medicine Discovery Center Identifier. Chemical structures and more information on all compounds tested in these studies are available at reference 18 as TCMDC IDs (Tres Cantos Medicine Discovery Center Identifiers).

	TCMDC ID	CI		TCMDC ID	CI		TCMDC ID	CI	
	**143101**	**A**		**143093**	**B**		**143407**	**D**	
	**143113**	**A**		**143094**	**B**		**143144**	**F**	
	**143122**	**A**		**143095**	**B**		**143168**	**F**	
	**143133**	**A**		**143211**	**C**		**143427**	**F**	
	**143557**	**A**		**143212**	**C**		**143164**	**G**	
	**143558**	**A**		**143213**	**C**		**143166**	**G**	
	**143570**	**A**		**143214**	**C**		**143517**	**G**	
	**143584**	**A**		**143216**	**C**		**143139**	**H**	
	**143639**	**A**		**143217**	**D**		**143140**	**H**	
	**143090**	**B**		**143218**	**D**		**143141**	**H**	
	**143091**	**B**		**143404**	**D**				
	**143092**	**B**		**143406**	**D**				

### Comparison AM/MAC / INF outputs

The comparison of the results obtained for the 88 compounds selected using the AM/MAC output and the corresponding 73 compounds using the INF output was carried out in order to investigate the correlation between the two outputs. Full data set for the two outputs, including information on the group and comments, was available for 68 compounds and the results obtained are shown in S8 Table. Correlation was considered “excellent” for those compounds included clearly in the same group (group 1, 2 or 3) independent of the output applied, “good” for those compounds showing agreement between the two outputs although not presenting a clear decision for one of the outputs and “poor” for those compounds included clearly in different groups for the two outputs. According to this rule, 59 compounds (87%) showed “excellent” correlation, 6 compounds (9%) presented “good” correlation and 3 compounds (4%) were included in the “poor” correlation group.

### Effect of the serum type on pEC50

The effect of the serum used as medium supplement on compound potency was investigated by comparing the pEC50 values at 96h obtained in the presence of horse serum, as described in the present work, with those previously obtained with foetal bovine serum [18]. For the whole group of 176 compounds, the pEC50s values were generally higher when determined in FBS: 51% higher in FBS *vs* 20% higher in HS (AM/MAC output) and 49% higher in FBS *vs* 15% higher in HS (INF output). However, when the comparison was limited to the selected hits, the really interesting compounds, this difference almost disappeared, with 35% of the pEC50 values higher in FBS and 27% higher in HS for AM/MAC output and 33% higher in FBS and 26% higher in HS for INF output. These results are shown in Table 4.

**Table 4:** Effect of the serum used in the assay on the pEC50 values obtained. Second and third columns represent data obtained for the AM/MAC output and for the INF output respectively. Data shown in the upper four rows correspond to all the compounds tested (*Leishmania donovani* box) while the ones shown in the lower four rows correspond to the selected sets of compounds (88 compounds for the AM/MAC output and 73 compounds for the INF output).

**FBS vs HS (AM/MAC & INF) for initial compounds**	**AM/MAC**	**INF**
	**176 cmpds**	**176 cmpds**
pIC50_96h_ FBS—pIC50_96h_ HS > 0.1	89 (51%)	86 (49%)
pIC50_96h_ FBS—pIC50_96h_ HS (-0.1/0.1)	51 (29%)	63 (36%)
pIC50_96h_ FBS—pIC50_96h_ HS < 0.1	36 (20%)	27 (15%)
**FBS vs HS (AM/MAC & INF) for selected compounds**	**AM/MAC**	**INF**
	**88 cmpds**	**73cmpds**
pIC50_96h_ FBS—pIC50_96h_ HS > 0.1	31 (35%)	24 (33%)
pIC50_96h_ FBS—pIC50_96h_ HS (-0.1/0.1)	33 (37%)	30 (41%)
pIC50_96h_ FBS—pIC50_96h_ HS < 0.1	24 (27%)	19 (26%)

## Discussion

### “Rate of action” assay to prioritise hits against *L*. *donovani*

Prioritisation of the most promising hits derived from screening campaigns at early stages of the drug discovery process, usually at the “Hit Optimization” or “Hit to Lead” level, is a crucial step to selectively accelerate the progression of “best in class” compounds. In fact, this step is expected to increase the probabilities of success while reducing the time and efforts needed to identify a candidate compound for development. Thus, the identification of new assays able to prioritise those compounds presenting a favourable property amongst a set of promising hits would represent a very important opportunity for drug discovery.

GSK has recently published the results of three screening campaigns leading to the assembly of three compound boxes active against the kinetoplastid parasites *T*. *brucei*, *T*. *cruzi* and *L*. *donovani* [18]. Among the parameters used in drug discovery for the selection of hit compounds, the rate of action is an important factor to take into account. This property is strongly linked to the mode of action of each compound and deserves special attention at an early stage of the drug discovery process. In the *in vitro* intra-macrophage assay here presented, amphotericin B and miltefosine, two of the currently available drugs against visceral leishmaniasis, showed different *in vitro* rate of action, with amphotericin B acting faster than miltefosine. For this reason, in the present work, the rate of action of these drugs was used to analyse and cluster compounds of the “*L*. *donovani* box”. The assay here presented was adapted from the *in vitro* replicative assay recently described by our group [19]. The potency of each compound was evaluated every 24 hours up to 96 hours and two different outputs, number of amastigotes per macrophage (AM/MAC) and percentage of infected cells (INF), were used to calculate pEC50s at each time point. As it has been previously demonstrated that the replication rate of intracellular amastigotes infecting differentiated THP-1 lies around two days [19], 24-hour time lapses appeared to be enough to study the evolution of pEC50 over time. Analysis of the potency data for compounds of the “*L*. *donovani* box” led, after application of demanding potency, selectivity and drug-like filters, to the identification of two hit sets that were progressed to biological and chemical clustering. The final aims were, on one hand, the prioritization of fast-acting compounds within these compound sets and, on the other hand, the validation of this assay by comparison of the results obtained with the biological and chemical clusterings.

### Biological clustering of selected compounds using AM/MAC and INF outputs

The classification of compounds from the two outputs according to the rate of action was carried out by comparing the pEC50 data at different time points for each compound with those obtained with AB and MF. The criteria used for the classification were set up on the basis of two experimental data: the difference of pEC50 values between 96h and 24h (Δ_96h-24h_), and the difference of pEC50 values between 96h and 48h (Δ_96h-48h_). This exercise identified, for the AM/MAC output, 64 (or 73%) compounds in group 1, 17 (or 19%) compounds in group 2 and 7 (or 8%) compounds in group 3. For the INF output, 49 (or 67%) compounds were in group 1, 13 (or 18%) compounds in group 2 and 11 (or 15%) compounds in group 3.

Most of the selected hits were characterized as fast-acting compounds probably due to the high value of the hits included in the “*L*. *donovani* box”, that were selected after application of very stringent potency and selectivity filters. However, the fact that still some slow acting compounds were identified (group 3), demonstrates the power of this assay to identify this class of compounds. In order to further prioritise these hits from group 1, standard filters could be applied (higher pIC50 values, more favourable physico-chemical properties, chemical availability, etc…).

### Comparison between biological clustering for AM/MAC and INF outputs

The pEC50 values at 96 hours showed a small but clear advantage for AM/MAC over INF input. The population of the different groups showed similar results for both outputs: group 1, was the most populated one (73% for AM/MAC and 67% for INF), followed by the intermediate group 2 (19% and 18%) and, finally, group 3 containing the slow-acting compounds (8% and 15%).

S8 Table shows results for biological compound clustering for AM/MAC and INF outputs. Sixty-eight compounds were common to the two outputs. The vast majority of the studied compounds (59 hits / 87%) showed “excellent” correlation, while it was assigned as “good” or “poor” for 6 (9%) and 3 compounds (4%) respectively. This result seems to reflect the high quality of the compounds included in the “*L*. *donovani* box”.

### Chemical clustering of selected compounds using AM/MAC and INF outputs

**Fig 3:**
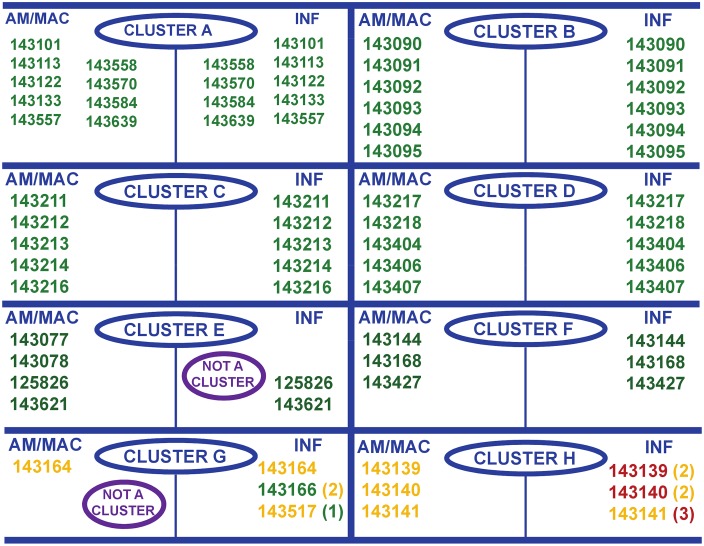
Comparison of the results obtained for the biological grouping and the chemistry clustering exercise using AM/MAC and INF outputs for the selected compounds. Compound TCMDC IDs (Tres Cantos Medicine Discovery Center Identifiers): green (group 1), orange (group 2) and red (group 3). Chemical structures for all compounds tested in these studies are available at reference 18 as TCMDC IDs (Tres Cantos Medicine Discovery Center Identifiers).

Once all compounds were classified through the biological clustering exercise, the validity of the rate of action assay and the information extrapolated were determined comparing the biological results to the chemical clustering. The rate of action is a property directly linked to the compound target or mechanism of action; thus, compounds acting through the same mechanism of action are also expected to present similar rate of action and should be classified in the same group by the assay here described. All compounds belonging to the same chemical class are expected to act through the same mechanism of action and should, therefore, elicit a response with a similar rate of action. In order to validate the biological results, we ran a chemical clustering exercise using the two groups of compounds previously classified through the biological clustering: the 88 high-quality hits identified within the AM/MAC output and the 73 identified within the INF output. In both cases, seven chemical scaffolds or clusters were obtained, comprising 35 and 34 compounds respectively. The comparison of the two analysis (Fig 3) showed that six of the seven chemical scaffolds independently identified by the two outputs were not only identical, but also included exactly the same 31 compounds, even though the two groups contained different number of compounds, thus confirming the robustness of the assay. In addition, the only different chemical scaffold for each output (clusters G and E) was represented in the other output by either one or two compounds, an insufficient number to be considered a chemical cluster, e.g. compound TCMDC-143164 in the AM/MAC output and compounds TCMDC-125826 and TCMDC-143621 in the INF output. The reason why less than half of the compounds (35 out of 88 for AM/MAC and 34 out of 73 for INF) were included in a cluster resides in the remarkable chemical diversity represented within the “*L donovani* box”. Thanks to the high-quality of the hits included in this box, seven chemical clusters per output were identified and analysed, a number that, in our opinion, is enough to validate the rate of action assay here described.

### Comparison chemical and biological clustering for AM/MAC and INF outputs

Even though the evaluation of the rate of action (biological clustering) and the determination of the minimum chemical scaffold (chemical clustering) offer different information for every compound, they are both linked to the compound mode of action. Thus, it is expected that compounds belonging to the same chemical class will also be assigned to the same “rate of action” group as defined in the present work. Comparison of the results shown in Fig 3 confirm this point, as compounds from five of the six chemical clusters identified for AM/MAC and INF (clusters A-D, and F) presented all their members in the same group (group 1) according to the biological clustering. In addition, the 2 compounds identified by the INF output belonging to cluster E in AM/MAC, TCMDC-125826 and TCMDC-143621, were also classified in group 1 by the biological clustering. Clusters G in INF and H in AM/MAC and INF deserve a special comment as the results suggest that these clusters are in the borderline of two groups. The INF output shows, for cluster G, one compound (TCMDC-143166) in group 1 although very close to the limit 1/2, one compound (TCMDC-143517) in group 2 although very close to the limit 1/2 and one compound (TCMDC-143164) in group 2, suggesting the cluster to present a rate of action close to the limit between groups 1 and 2. The only compound of this series identified by AM/MAC, TCMDC-143164, was assigned to group 2, in good agreement with this suggestion. Concerning cluster H, the analysis of the AM/MAC output assigned the three compounds to group 2 while INF assigned two of them, TCMDC-143139 and TCMDC-143140, to group 3 although very close to limit 2/3 too and one of them, TCMDC-143141, to group 2 although very close to limit 2/3; it can be concluded that cluster H presents a rate of action in the frontier between groups 2 and 3. In summary, an excellent correlation was observed between the biological assay and the chemical clustering exercise as well as between the two outputs used for those compounds clearly included in group 1 or, in other words, the most promising compounds. For compounds belonging to groups 2 and 3, the assay was also able to identify chemical clusters in the borderline of two groups. These results clearly demonstrate the validity of this time-to-kill assay to prioritise and progress the most promising hits obtained in an HTS campaign on the basis of the rate of action.

### Conclusion

In conclusion, the assay here described allowed the grouping of the compounds of the “*L*. *donovani* box” according to their rate of action, defined in comparison with standard drugs amphotericin B and miltefosine. It was possible, in fact, to discriminate between compounds faster, similar or slightly slower than fast-acting amphotericin B; faster, similar or slightly slower than slow-acting miltefosine but slower than amphotericin B; and slower than both drugs. Cross-comparison of the biological and chemical clustering for these compounds revealed that molecules belonging to the same chemical series showed the same behaviour in terms of rate of action. In particular, five chemical series were characterized as belonging to group 1 when analysed using the AM/MAC or INF outputs. These series contain very promising compounds that deserve further studies by the scientific community. These data altogether confirm this assay can provide very useful hints on the rate of action of new compounds and, consequently, can be used for the prioritization of the most promising new chemical series within a group of hits from screening. Likewise, it validates the tactics for the selection of hit series based on joint chemical and biological clustering.

## Supporting information

S1 Table pEC50 values obtained for the 176 “*L*. *donovani* box” hits evaluated with the rate of action assay using AM/MAC output at four time points (24, 48, 72 and 96 hours). The pEC50 numbers represent the average of two assay runs. pEC50 = -log EC_50_ (M). TCMDC ID: Tres Cantos Medicine Discovery Center Identifier. Chemical structures and more information on all compounds tested in these studies are available at reference 18 as TCMDC IDs (Tres Cantos Medicine Discovery Center Identifiers).
(PDF)

S2 Table pEC50 values obtained for the 176 “*L*. *donovani* box” hits evaluated with the rate of action assay using INF output at four time points (24, 48, 72 and 96 hours). The pEC50 numbers represent the average of two assay runs. pEC50 = -log EC_50_ (M). TCMDC ID: Tres Cantos Medicine Discovery Center Identifier Chemical structures and more information on all compounds tested in these studies are available at reference 18 as TCMDC IDs (Tres Cantos Medicine Discovery Center Identifiers).
(PDF)

S3 Table Z’ values calculated for the two outputs (AM/MAC and INF) at each time point. (PDF)

S4 Table pEC50 values obtained for the 88 “*L*. *donovani* box” selected hits evaluated with the rate of action assay using AM/MAC output at four time points (24, 48, 72 and 96 hours). The pEC50 numbers represent the average of two assay runs. pEC50 = -log EC_50_ (M). TCMDC ID: Tres Cantos Medicine Discovery Center Identifier. Chemical structures and more information on all compounds tested in these studies are available at reference 18 as TCMDC IDs (Tres Cantos Medicine Discovery Center Identifiers).
(PDF)

S5 Table pEC50 values obtained for the 73 “*L*. *donovani* box” selected hits evaluated with the rate of action assay using INF output at four time points (24, 48, 72 and 96 hours). The pEC50 numbers represent the average of two assay runs. pEC50 = -log EC_50_ (M). TCMDC ID: Tres Cantos Medicine Discovery Center Identifier. Chemical structures and more information on all compounds tested in these studies are available at reference 18 as TCMDC IDs (Tres Cantos Medicine Discovery Center Identifiers).
(PDF)

S6 Table Rate of action classification of the 88 selected compounds with the AM/MAC output and the reference drugs (R). Δ96h-24h = pEC50_96h_-pEC50_24h_; Δ96h-48h = pEC50_96h_-pEC50_48h_; Gr = group assigned; Gr* = alternative group. The alternative group reflects cases where the pEC50 difference is very close to the limit value (± 0.2 for limit 1/2 and ± 0.15 for limit 2/3). TCMDC ID: Tres Cantos Medicine Discovery Center Identifier. Chemical structures and more information on all compounds tested in these studies are available at reference 18 as TCMDC IDs (Tres Cantos Medicine Discovery Center Identifiers).
(PDF)

S7 Table Rate of action classification of the 73 selected compounds with the INF output and the reference drugs (R). Δ96h-24h = pEC50_96h_-pEC50_24h_; Δ96h-48h = pEC50_96h_-pEC50_48h_; Gr = group assigned; Gr* = alternative group. The alternative group reflects cases where the pEC50 difference is very close to the limit value (± 0.2 for limit 1/2 and for limit 2/3). TCMDC ID: Tres Cantos Medicine Discovery Center Identifier. Chemical structures and more information on all compounds tested in these studies are available at reference 18 as TCMDC IDs (Tres Cantos Medicine Discovery Center Identifiers).
(PDF)

S8 Table Comparison of the results from the biological grouping exercise obtained for the two selected sets of hits using the AM/MAC and INF outputs. Gr AM: group assigned using the AM/MAC output. pEC50 96h: pEC50 value obtained at 96 hours using the corresponding output (AM/MAC or INF). G2: alternative group assigned using the corresponding output (AM/MAC or INF). Agree: type of agreement obtained for a compound when comparing results from AM/MAC and INF outputs; it was defined as Excellent (E), Good (G), Poor (P) or Not Applicable (NA) for those compounds for which there are not results for the two outputs. pEC50 = -log EC_50_ (M). TCMDC ID: Tres Cantos Medicine Discovery Center Identifier. Chemical structures and more information on all compounds tested in these studies are available at reference 18 as TCMDC IDs (Tres Cantos Medicine Discovery Center Identifiers).
(PDF)
