# Supplementary material for: Unravelling the rate of action of hits in the Leishmania donovani box using standard drugs amphotericin B and miltefosine
Source: PLoS Negl Trop Dis. 2017 May 25;11(5):e0005629. doi: 10.1371/journal.pntd.0005629 (PMC5462473; doi:10.1371/journal.pntd.0005629)
Supplement: S1 Table — The pEC50 numbers represent the average of two assay runs. pEC50 = -log EC50 (M). TCMDC ID: Tres Cantos Medicine Discovery Center Identifier. Chemical structures and more information on all compounds tested in these studies are available at reference 18 as TCMDC IDs (Tres Cantos Medicine Discovery Center Identifiers). (PDF) [file pntd.0005629.s001.pdf]

TCMDC ID	AM/MAC AVG 24h	AM/MAC AVG 48h	AM/MAC AVG 72h	AM/MAC AVG 96h
124508	<5	<5	5.07	5.37
125160	<5	<5	<5	6.44
125387	<5	5.05	5.33	5.45
125826	5.47	5.60	5.74	5.81
142704	5.15	5.35	5.49	5.53
142900	6.04	6.03	6.07	6.12
143075	<5	<5	<5	<5
143077	5.65	5.66	5.89	6.02
143078	<5	5.09	5.11	5.16
143086	<5	<5	5.21	5.30
143090	5.25	5.39	5.39	5.54
143091	5.28	5.29	5.39	5.44
143092	5.64	5.72	5.76	5.97
143093	5.21	5.23	5.32	5.39
143094	5.28	5.39	5.40	5.47
143095	5.41	5.42	5.54	5.65
143096	5.37	5.35	5.43	5.44
143098	<5	<5	<5	<5
143099	<5	<5	<5	<5
143101	6.22	6.28	6.38	6.39
143106	ND	5.21	5.68	5.71
143110	<5	<5	5.19	5.22
143113	5.94	6.07	5.97	6.04
143115	<5	<5	5.18	5.34
143117	<5	5.24	5.51	5.63
143119	<5	<5	<5	<5
143122	6.12	6.14	6.18	6.21
143124	<5	5.02	<5	5.14
143129	<5	5.04	5.30	5.37
143133	6.42	6.46	6.76	6.58
143136	<5	<5	6.01	6.05
143139	<5	5.03	5.29	5.29
143140	<5	<5	5.06	5.14
143141	<5	5.03	5.18	5.35
143144	5.50	5.59	5.67	5.73
143145	5.38	5.51	5.65	5.52
143147	<5	<5	5.16	5.37
143163	5.89	6.05	6.07	6.10
143164	<5	5.66	6.13	5.82
143165	<5	<5	<5	5.04
143166	5.05	5.39	5.41	5.39
143168	6.23	6.34	6.48	6.55
143169	<5	5.43	5.07	5.55
143170	<5	5.23	5.40	5.38
143171	<5	5.00	5.95	5.98
143174	<5	<5	5.57	5.53
143175	<5	5.11	5.26	5.37
143180	6.26	6.47	6.57	6.58
143181	5.24	5.21	5.62	5.83
143184	<5	<5	<5	<5
143188	<5	<5	<5	5.04
143196	<5	<5	5.16	5.40
143197	<5	<5	<5	5.50
143201	<5	<5	<5	<5
143202	<5	<5	<5	<5
143208	<5	<5	5.03	5.11
143211	5.71	5.76	5.84	5.93
143212	6.14	6.29	6.36	6.35
143213	6.54	6.69	6.69	6.86
143214	6.11	6.21	6.26	6.26

TCMDC ID	AM/MAC AVG 24h	AM/MAC AVG 48h	AM/MAC AVG 72h	AM/MAC AVG 96h
143216	6.10	6.23	6.29	6.36
143217	5.80	5.96	6.02	6.05
143218	5.98	6.14	6.20	6.19
143223	<5	<5	<5	<5
143236	5.49	5.69	5.75	5.70
143237	<5	<5	<5	<5
143239	<5	<5	<5	5.63
143245	<5	<5	<5	<5
143246	<5	<5	<5	<5
143249	<5	<5	<5	<5
143252	<5	5.10	5.37	5.37
143255	<5	<5	<5	<5
143259	<5	<5	<5	<5
143260	<5	<5	6.02	6.16
143261	5.32	5.48	5.53	5.54
143266	<5	<5	<5	<5
143268	<5	<5	<5	<5
143269	<5	5.19	5.23	5.34
143271	<5	<5	ND	<5
143274	<5	<5	5.18	5.28
143277	<5	<5	<5	5.01
143278	<5	<5	5.10	5.15
143280	<5	<5	<5	<5
143281	5.02	5.78	6.17	6.25
143285	<5	<5	<5	5.66
143287	<5	<5	<5	<5
143296	5.33	5.66	5.80	5.95
143297	5.40	5.74	5.87	5.95
143305	5.70	5.87	5.92	5.93
143306	<5	<5	<5	<5
143315	5.12	5.55	5.70	5.76
143327	<5	5.92	6.09	6.83
143340	<5	<5	<5	<5
143344	<5	5.34	5.43	5.41
143345	<5	<5	5.27	5.19
143347	<5	5.74	5.87	5.92
143348	<5	<5	<5	<5
143349	<5	<5	<5	<5
143350	5.37	5.63	5.77	5.88
143351	<5	5.44	5.57	5.68
143353	5.02	5.57	5.12	5.14
143355	<5	<5	<5	<5
143358	<5	5.10	6.11	6.53
143367	<5	<5	<5	<5
143375	<5	<5	5.17	5.16
143383	<5	<5	5.33	5.84
143388	<5	<5	<5	<5
143391	<5	5.43	5.76	5.95
143396	<5	5.21	5.45	6.01
143397	<5*	<5	<5	5.15
143398	<5	5.51	5.78	5.98
143404	5.69	5.73	5.80	5.84
143406	6.01	6.26	6.31	6.38
143407	5.38	5.54	5.50	5.66
143418	5.06	5.24	5.32	5.41
143419	<5	5.16	5.26	5.43
143427	5.36	5.41	5.53	5.47
143431	<5	5.16	5.26	5.33
143441	<5	<5	<5	<5
143442	<5	<5	<5	5.36

TCMDC ID	AM/MAC AVG 24h	AM/MAC AVG 48h	AM/MAC AVG 72h	AM/MAC AVG 96h
143443	5.16	5.42	5.53	5.58
143447	5.76	6.03	5.94	6.18
143448	5.08	5.24	5.41	5.33
143450	<5	<5	<5	<5
143451	5.29	5.37	5.59	5.76
143459	6.42	6.70	6.91	6.99
143473	<5	<5	<5	5.46
143478	5.34	5.44	5.60	5.61
143480	<5	<5	<5	5.33
143482	<5	<5	<5	<5
143483	<5	5.03	5.48	5.78
143486	5.96	6.05	6.11	6.15
143489	5.48	5.69	5.74	5.73
143491	<5	5.04	5.14	5.16
143501	5.87	6.01	6.11	6.14
143503	5.68	5.81	5.86	5.96
143508	5.67	5.68	5.70	5.75
143509	5.54	5.60	5.62	5.66
143514	<5	5.27	5.29	5.38
143517	5.25	5.44	5.47	5.51
143518	6.14	6.33	6.35	6.46
143521	5.41	5.68	5.77	5.85
143522	<5	<5	5.52	5.11
143523	<5	<5	<5	<5
143524	6.13	6.29	6.34	6.34
143531	<5	<5	<5	<5
143532	<5	<5	<5	<5
143534	5.42	5.51	5.50	5.55
143536	<5	<5	<5	<5
143538	<5	<5	5.57	6.01
143554	<5	<5	5.41	5.15
143557	5.33	5.32	5.38	5.52
143558	6.08	6.21	6.23	6.32
143563	<5	5.52	5.56	5.56
143566	<5	<5	<5	<5
143567	<5	<5	<5	<5
143568	5.53	5.68	5.71	5.84
143570	5.96	6.15	6.24	6.33
143571	<5	<5	5.12	5.00
143573	<5	<5	5.02	5.12
143574	<5	<5	5.08	5.26
143576	<5	<5	<5	<5
143577	5.06	5.08	5.17	5.00
143584	5.79	6.06	6.10	6.19
143586	5.93	6.02	5.95	5.98
143591	<5	<5	5.59	5.67
143594	<5	<5	<5	<5
143600	5.58	5.70	5.77	5.80
143603	6.15	6.15	6.17	5.76
143607	5.48	5.88	5.82	5.86
143618	<5	<5	<5	<5
143621	5.77	5.86	5.96	5.96
143628	5.10	6.03	5.25	6.19
143633	5.62	5.72	5.88	6.00
143639	5.11	5.28	5.35	5.29
143647	5.60	5.72	5.72	5.77
Miltefosine	5.31	6.05	6.22	6.28
Ampho B	6.87	7.11	7.27	7.16
