# Supplementary material for: Unravelling the rate of action of hits in the Leishmania donovani box using standard drugs amphotericin B and miltefosine
Source: PLoS Negl Trop Dis. 2017 May 25;11(5):e0005629. doi: 10.1371/journal.pntd.0005629 (PMC5462473; doi:10.1371/journal.pntd.0005629)
Supplement: S3 Table — (PDF) [file pntd.0005629.s003.pdf]

Z' values		
T	AM/MAC	INF
24h	0.72 ± 0.07	0.87 ± 0.04
48h	0.7 ± 0.08	0.88 ± 0.03
72h	0.61 ± 0.07	0.87 ± 0.02
96h	0.58 ± 0.1	0.83 ± 0.04
