# Supplementary material for: Unravelling the rate of action of hits in the Leishmania donovani box using standard drugs amphotericin B and miltefosine
Source: PLoS Negl Trop Dis. 2017 May 25;11(5):e0005629. doi: 10.1371/journal.pntd.0005629 (PMC5462473; doi:10.1371/journal.pntd.0005629)
Supplement: S7 Table — Δ96h-24h = pEC5096h-pEC5024h; Δ96h-48h = pEC5096h-pEC5048h; Gr = group assigned; Gr* = alternative group. The alternative group reflects cases where the pEC50 difference is very close to the limit value (± 0.2 for limit 1/2 and for limit 2/3). TCMDC ID: Tres Cantos Medicine Discovery Center Identifier. Chemical structures and more information on all compounds tested in these studies are available at reference 18 as TCMDC IDs (Tres Cantos Medicine Discovery Center Identifiers). (PDF) [file pntd.0005629.s007.pdf]

TCMDC ID	Δ96h-24h	Δ96h-48h	Gr	TCMDC ID	Δ96h-24h	Δ96h-48h	Gr	Gr'	TCMDC ID	Δ96h-24h	Δ96h-48h	Gr	Gr'
125826	0.11	0.00	1	143305	0.23	0.13	1		143164	>1.3	0.20	2	
142900	0.04	0.03	1	143350	0.28	0.11	1		143252	>0.92	0.31	2	
143090	0.09	0.04	1	143404	0.02	0.07	1		143274	>0.78	0.39	2	
143091	-0.04	0.01	1	143406	0.26	0.13	1		143297	1.16	0.21	2	
143092	0.14	0.12	1	143407	0.20	0.16	1		143315	>1.35	0.28	2	
143093	0.00	0.06	1	143427	0.10	0.04	1		143391	>1.23	0.49	2	
143094	0.08	0.29	1	143447	0.22	0.21	1		143398	>1.38	0.34	2	
143095	0.02	0.13	1	143459	0.42	0.15	1		143633	>1.5	0.25	2	
143101	0.06	0.13	1	143486	0.20	0.06	1		143141	>0.87	0.66	2	or 3
143113	0.17	0.04	1	143489	0.26	0.05	1		143196	>0.89	0.65	2	or 3
143122	0.08	0.08	1	143491	0.25	0.15	1		143351	>1.27	0.68	2	or 3
143133	0.18	0.16	1	143501	0.19	0.10	1		143431	>0.84	0.51	2	or 3
143144	0.10	0.04	1	143524	0.09	0.01	1		124508	>0.83	>0.83	3	or 2
143145	0.04	-0.05	1	143557	0.07	0.12	1		143139	>0.88	>0.88	3	or 2
143168	-0.04	0.04	1	143558	0.15	0.05	1		143140	>0.72	>0.72	3	or 2
143180	0.20	0.10	1	143570	0.31	0.20	1		143129	>0.95	>0.95	3	
143211	0.08	-0.01	1	143584	0.38	0.21	1		143174	>0.94	>0.94	3	
143212	0.18	0.09	1	143586	0.03	0.01	1		143327	>1.61	>1.61	3	
143213	0.12	0.05	1	143607	0.38	0.20	1		143344	>1.08	>1.08	3	
143214	0.14	0.09	1	143621	0.23	0.23	1		143345	>0.8	>0.8	3	
143216	0.11	0.10	1	143639	0.15	0.07	1		143347	>1.59	1.19	3	
143217	0.11	0.05	1	143647	0.15	0.04	1		143358	>1.09	>1.09	3	
143218	0.08	-0.02	1	143166	0.67	0.09	1	or 2	143591	>1.01	>1.01	3	
143236	0.06	-0.03	1	143296	0.68	0.16	1	or 2	Ampho B	0.2	0.04	1	
143261	0.17	0.06	1	143517	0.78	0.06	2	or 1	Miltefosine	1.04	0.52	2	
