# Supplementary material for: Unravelling the rate of action of hits in the Leishmania donovani box using standard drugs amphotericin B and miltefosine
Source: PLoS Negl Trop Dis. 2017 May 25;11(5):e0005629. doi: 10.1371/journal.pntd.0005629 (PMC5462473; doi:10.1371/journal.pntd.0005629)
Supplement: S8 Table — Gr AM: group assigned using the AM/MAC output. pEC50 96h: pEC50 value obtained at 96 hours using the corresponding output (AM/MAC or INF). G2: alternative group assigned using the corresponding output (AM/MAC or INF). Agree: type of agreement obtained for a compound when comparing results from AM/MAC and INF outputs; it was defined as Excellent (E), Good (G), Poor (P) or Not Applicable (NA) for those compounds for which there are not results for the two outputs. pEC50 = -log EC50 (M). TCMDC ID: Tres Cantos Medicine Discovery Center Identifier. Chemical structures and more information on all compounds tested in these studies are available at reference 18 as TCMDC IDs (Tres Cantos Medicine Discovery Center Identifiers). (PDF) [file pntd.0005629.s008.pdf]

TCMDC ID	Gr. AM	pEC50 96h	G2	Agree	Gr. INF	pEC50 96h	G2
124508	3	5.37		E	3	5.13	2
125826	1	5.81		E	1	5.47	
142900	1	6.12		E	1	5.98	
143077	1	6.02		NA	NA	NA	NA
143078	1	5.16		NA	NA	NA	NA
143090	1	5.54		E	1	5.22	
143091	1	5.44		E	1	5.13	
143092	1	5.97		E	1	5.65	
143093	1	5.39		E	1	5.11	
143094	1	5.47		E	1	5.26	
143095	1	5.65		E	1	5.34	
143101	1	6.39		E	1	6.18	
143113	1	6.04		E	1	6.00	
143117	2	5.63		NA	NA	NA	
143122	1	6.21		E	1	6.17	
143129	2	5.40		P	3	5.26	
143133	1	6.58		E	1	6.57	
143136	3	6.05		NA	NA	NA	
143139	2	5.29		G	3	5.18	2
143140	2	5.14		G	3	5.02	2
143141	2	5.35		E	2	5.18	3
143144	1	5.73		E	1	5.48	
143145	1	5.52		E	1	5.39	
143147	2	5.37	3	NA	NA	NA	
143164	2	5.82		E	2	5.60	
143166	NA	NA		NA	1	5.38	2
143168	1	6.55		E	1	6.08	
143174	3	5.53		E	3	5.25	
143180	1	6.58		E	1	6.39	
143188	1	5.04	2	NA	NA	NA	
143196	3	5.40		G	2	5.19	3
143211	1	5.93		E	1	5.67	
143212	1	6.35		E	1	6.18	
143213	1	6.86		E	1	6.58	
143214	1	6.26		E	1	6.16	
143216	1	6.36		E	1	6.10	
143217	1	6.05		E	1	5.77	
143218	1	6.19		E	1	6.03	
143236	1	5.70		E	1	5.61	
143252	NA	NA		NA	2	5.22	
143261	1	5.54		E	1	5.42	
143269	1	5.30		NA	NA	NA	
143274	2	5.28	1	E	2	5.09	
143278	2	5.15	3	NA	NA	NA	
143281	2	6.20	3	NA	NA	NA	
143285	3	5.66		NA	NA	NA	
143296	1	5.95	2	E	1	5.70	2

TCMDC ID	Gr. AM	pEC50 96h	G2	Agree	Gr. INF	pEC50 96h	G2
143297	1	5.95	2	G	2	5.83	
143305	1	5.93		E	1	5.80	
143315	1	5.76	2	G	2	5.65	
143327	NA	NA		NA	3	5.92	
143344	2	5.41		P	3	5.38	
143345	2	5.19		G	3	5.10	2
143347	2	5.92		P	3	5.90	
143350	1	5.88		E	1	5.47	
143351	2	5.68		E	2	5.58	3
143358	3	6.53		E	3	5.39	
143375	1	5.16		NA	NA	NA	
143391	2	5.95	3	E	2	5.54	
143398	2	5.98	3	E	2	5.68	
143404	1	5.84		E	1	5.62	
143406	1	6.38		E	1	6.23	
143407	1	5.66		E	1	5.46	
143418	1	5.41		NA	NA	NA	
143427	1	5.47		E	1	5.34	
143431	2	5.33		E	2	5.14	3
143443	1	5.58		NA	NA	NA	
143447	1	6.18		E	1	5.92	
143451	1	5.80		NA	NA	NA	
143459	1	6.99		E	1	6.71	
143478	1	5.61		NA	NA	NA	
143486	1	6.15		E	1	6.09	
143489	1	5.73		E	1	5.49	
143491	1	5.16		E	1	5.11	
143501	1	6.14		E	1	5.99	
143503	1	6.00		NA	NA	NA	
143508	1	5.75		NA	NA	NA	
143517	NA	NA		NA	2	5.41	1
143518	1	6.50		NA	NA	NA	
143524	1	6.34		E	1	6.15	
143557	1	5.52		E	1	5.34	
143558	1	6.32		E	1	6.09	
143568	1	5.84		NA	NA	NA	
143570	1	6.33		E	1	6.20	
143584	1	6.19		E	1	6.07	
143586	1	5.98		E	1	5.92	
143591	3	5.67		E	3	5.31	
143600	1	5.80		NA	NA	NA	
143607	1	5.86		E	1	5.54	
143621	1	5.96		E	1	5.90	
143633	NA	NA		NA	2	5.80	
143639	1	5.29		E	1	5.16	
143647	1	5.77		E	1	5.66	
